# First-in-human study of alpibectir (BVL-GSK098), a novel potent anti-TB drug

**DOI:** 10.1093/jac/dkae107

**Published:** 2024-04-24

**Authors:** Michel Pieren, Ana Abáigar Gutiérrez-Solana, Rosa María Antonijoan Arbós, Gary W Boyle, Myriam Davila, Maria Davy, Marc Gitzinger, Lisa Husband, María S Martínez-Martínez, Dolores Ochoa Mazarro, Eleni Pefani, Sophie L Penman, Modesto J Remuiñán, Georgios Vlasakakis, Markus Zeitlinger, Glenn E Dale

**Affiliations:** BioVersys AG, Basel, Switzerland; CTI Laboratory Services S.L., Ibaizabal bidea, 702, 48160 Derio, Biscay, Spain; Institut de Recerca de l'HSCSP, IIB Sant Pau, Centre d'Investigació de Medicaments (CIM), Barcelona, Spain; GSK, Stevenage, UK; BioVersys AG, Basel, Switzerland; GSK, Stevenage, UK; BioVersys AG, Basel, Switzerland; BioVersys SAS, Lille, France; BioVersys SAS, Lille, France; GSK, Tres Cantos, Spain; Clinical Trials Unit, Clinical Pharmacology Department, Facultad de Medicina, Hospital Universitario de La Princesa, Universidad Autónoma de Madrid (UAM), Instituto de Investigación Sanitaria La Princesa (IP), Madrid, Spain; GSK, Stevenage, UK; GSK, London, UK; GSK, Tres Cantos, Spain; GSK, Philadelphia, PA, USA; Department of Clinical Pharmacology, Vienna University Hospital, Medical University of Vienna, Vienna, Austria; BioVersys AG, Basel, Switzerland; BioVersys SAS, Lille, France

## Abstract

**Background:**

The clinical candidate alpibectir augments the activity of, and overcomes resistance to, the anti-TB drug ethionamide *in vitro* and *in vivo*.

**Objectives:**

A Phase 1, double-blind, randomized, placebo-controlled study to investigate the safety, tolerability, pharmacokinetics (PK) and food effect of alpibectir administered as single and multiple oral doses in healthy volunteers (NCT04654143).

**Methods:**

Eighty participants were randomized. In single ascending dose (SAD), a total of six dose levels of alpibectir (0.5 to 40 mg) were tested under fasted and fed (10 mg) conditions as single daily doses in sequential cohorts. In multiple ascending dose (MAD), repeat doses (5 to 30 mg) were administered once daily for 7 days in three sequential cohorts.

**Results:**

No serious adverse event was reported. Thirteen participants across groups experienced a total of 13 mild or moderate treatment-emergent adverse events. Alpibectir showed rapid absorption after single dose (mean *T*_max_ range of 0.88 to 1.53 h). Food affected the PK of alpibectir, characterized by a slower absorption (mean *T*_max_ 3.87 h), a lower *C*_max_ (−17.7%) and increased AUC_0–*t*_ (+19.6%) compared with the fasted condition. Following repeat dosing, dose proportionality was shown for both *C*_max_ and AUC_0–tau_. Accumulation of alpibectir was observed across all doses, with a more profound effect on AUC during a dosing interval (AUC_0–tau_) compared with *C*_max_ (1.8- and 1.3-fold on average), respectively. Steady state was considered to have been achieved by Day 7 of dosing.

**Conclusions:**

Alpibectir was generally well tolerated, and no clinically relevant safety findings were identified in the participants treated during SAD or MAD. The PK is dose-proportional and affected by food.

## Introduction

Despite being a curable disease, TB remains a serious global public health challenge. According to the WHO’s 2022 Global TB report, an estimated 10.6 million people fell ill with TB in 2021—an increase of 4.5% from 2020—and 1.6 million people died from TB.^1^ The burden of drug-resistant TB (DR-TB) also increased by 3% between 2020 and 2021, with 450 000 new cases of rifampicin-resistant TB. This is the first time in many years an increase has been reported in the number of people affected with TB. TB services were among many others disrupted by the COVID-19 pandemic in 2021, and the impact on the TB response has been particularly severe. Ongoing conflicts across Eastern Europe, Africa and the Middle East, and undernutrition have further exacerbated the situation for vulnerable populations.^1,2^

Issues with current combination treatment regimens for DR-TB include length of treatment (6 to 18 months), significant side effects, emergence of drug resistance,^3^ and drug–drug interactions. Drugs recently approved for the treatment of DR-TB by the FDA and the EMA include bedaquiline, delamanid and pretomanid^4–6^; they are currently being evaluated for the treatment of drug-susceptible TB as well.^7^ Resistance to bedaquiline and delamanid emerged soon after their introduction in the clinic, with reports of resistance first *in vitro* and then clinically.^8–11^ Thus, there is an urgent need to develop effective, affordable, accessible and well-tolerated new therapies with novel modes of action to create regimens that can fill the gaps in current TB treatment.

Ethionamide (Eto) was first synthesized in 1956 and has been used for the treatment of DR-TB for several decades. Eto is a prodrug that must be bioactivated inside *Mycobacterium tuberculosis* to exert its antitubercular activity. For a long time, it was assumed that this bioactivation occurred only through the mycobacterial monooxygenase EthA, which is strongly controlled by the transcriptional repressor EthR.^12,13^ Consequently, high doses of Eto, to the limit of acceptable tolerability, are required to achieve a satisfactory efficacy in humans resulting in dose-dependent adverse events, mainly gastrointestinal disturbances.^14,15^ More recently, alternative bioactivation pathways of Eto have been identified, mediated by the enzymes EthA2 and MymA. Both pathways are under the control of the transcription regulators EthR2 and VirS, respectively.^16,17^ Notably, compounds targeting EthR2 (SMARt420) or VirS (SMARt751) have been described, capable of stimulating the expression of the alternative bioactivation pathways.^17,18^

Alpibectir (also known as BVL-GSK098) is an optimized version of SMARTt420 and SMARt751 and the first example of a small molecule targeting a mycobacterial transcription regulator in clinical development, a ground-breaking approach with the potential to create a paradigm shift in the treatment options for TB. Alpibectir acts on VirS, thereby increasing the level of bioactivation of Eto, both *in vitro* and *in vivo*. By stimulating the alternative MymA pathway, alpibectir overcomes Eto resistance due to EthA mutations.^19^ Based on existing data, alpibectir has the potential to lower the efficacious human oral dose of Eto by at least two thirds, from  ≥ 750 mg to  ≤ 250 mg (P. Gamallo and M.S. Martinez-Martinez, PBPK and PK/PD report, unpublished data), with the potential to significantly minimize dose-dependent side effects and improve patient compliance, allowing clinicians to exploit the full potential of this 60-year-old drug.

Alpibectir has a high solubility and passive permeability, and is not a P-glycoprotein or breast cancer resistant protein substrate and therefore showed rapid absorption and exhibited moderate to high oral bioavailability, ranging from 67% to complete across the non-clinical species (mouse, rat and dog) studied. *In vitro* data suggested that alpibectir protein binding is low (<80%) in non-clinical species and humans, in agreement with observed moderate volume of distribution in preclinical species (I. Saiz-Nicolas, *in vitro* ADME and *in vivo* DMPK reports, unpublished data). The No Observed Adverse Effect Level (NOAEL) in dogs was at the highest tested dose of 15 mg/kg/d, with a sex-averaged *C*_max_ of 4520 ng/mL (Remuiñán *et al.*, unpublished data). Respecting a safety factor of 10 and similar free fraction between dogs and human, the maximum exposure safety threshold for the first-in-human (FIH) trial was set to a total plasma *C*_max_ of 452 ng/mL.

The FIH study was designed to assess the safety, tolerability and pharmacokinetics (PK) of single and repeated oral doses of alpibectir in healthy adult participants. In addition, a food effect assessment was also conducted to investigate the influence of food on the PK of alpibectir. The results of this study are intended to be used to identify appropriate and well-tolerated doses of alpibectir to be used in further studies.

## Materials and methods

### Study design and participants

This was a randomized, double-blind, placebo-controlled FIH study (clinicaltrials.gov registration number NCT04654143). The primary objectives of the study were to investigate the safety and tolerability of alpibectir after single (SAD) and multiple (MAD) oral ascending doses in healthy adult participants. The secondary objectives of the study were to: (i) determine the PK of single and repeated oral doses of alpibectir, (ii) assess the effect of food on the PK of alpibectir following a single oral dose, and (iii) examine the extent of accumulation and achievement of steady state following repeated oral doses of alpibectir. As an exploratory objective, plasma and urine samples were collected for the analysis of alpibectir-related material to elucidate routes of metabolism following (S. Thomas *et al.*, unpublished data) single and repeated oral administration. The results of this exploratory objective will be reported separately (manuscript submitted).

The study enrolled healthy male and female participants aged between 18 and 55 years, with a body weight of ≥50 or ≥45 kg (for male and female participants, respectively), a BMI of between 19 and 30 kg/m^2^, and no clinically signiﬁcant abnormalities in vital signs or ECGs or in chemistry, haematology or liver function tests.

In SAD, a total of six dose levels of alpibectir (0.5, 1.5, 4, 10, 25 and 40 mg) were tested in seven sequential cohorts. In MAD, a total of three dose levels of alpibectir (5, 14 and 30 mg) were tested in three sequential cohorts. Each cohort consisted of eight healthy participants and the participants were randomized in a 3:1 ratio to receive one single administration (SAD) or seven daily doses (MAD) of alpibectir (six participants/cohort) or placebo (two participants/cohort) under fasted conditions. The last cohort in SAD investigated the effect of food (high-fat, high-caloric meal according to FDA guidance^20^) on the PK of alpibectir administering a dose already tested in fasted conditions (10 mg).

Safety was assessed during the conduct of the study by physical examination, neurological examination, monitoring of vital signs (blood pressure, heart rate, body temperature, respiratory rate, etc.), 24 h Holter monitoring and recording of ECGs at predeﬁned time points. Laboratory safety assessments were done for the single-dosing cohorts on Day −1, at 24 and 72 h post-dose, and during the follow-up visit (Day 11 to 14) during the single-dosing period and for repeat dose cohorts on Day −1, in the morning (pre-dose) on Days 2, 3, 5 and 7, 8 and 10, and during the follow-up visit (Day 17 to 20). All adverse events (AEs) experienced during the conduct of the study were reported and assessed for their intensity and causality. Follow-up of AEs was conducted according to the protocol. No participant had concomitant medication other than paracetamol <2 g/d. Telemetry (Holter ECG) was recorded from 1 h pre-dose until 24 h post-dose on Day 1. ECGs were recorded at 72 h post-dose and at end of study (Day 11 to 14) during single dosing. For multiple dosing, continuous lead II ECG was monitored on Day 1 and on Day 7, from pre-dose until at least 8 h post-dose. ECGs were recorded pre-dose at Days 1, 2, 3, 5, 7, 8 and 10 and finally at the end of study (Day 17 to 20). All ECGs were taken as triplicate measurements.

For more detailed information, please refer to the Supplementary Information, available as Supplementary data at *JAC* Online.

### Plasma samples

Blood samples for plasma drug concentration quantiﬁcation analysis were collected at predeﬁned time points, which for SAD included pre-dose and 0.25, 0.5, 0.75, 1, 1.5, 2, 3, 4, 6, 8, 12, 15, 24, 36, 48 and 72 h after dosing on Day 1. For MAD, blood samples were collected at the same time points as the ones listed above for SAD on Days 1 (up to 24 h post-dose) and 7 (up to 72 h post-dose), including a pre-dose sample on Days 5 and 6. The sample taken at Day 8, 24 h after the last dose, was regarded as a pre-dose sample. Blood samples (1.8, 4.0 or 7.5 mL) were collected via an indwelling cannula (or vein puncture) into anticoagulant K_3_EDTA tubes and immediately placed on ice. After centrifugation (4°C, 1500 × **g**, for 10 minutes) aliquots from plasma samples were made and stored in a ≤−65°C temperature-controlled freezer.

### Bioanalytical methods for determination of alpibectir concentration in plasma

A robust and reproducible bioanalytical method was developed, validated and used for determination of plasma concentrations of alpibectir in human plasma. Plasma samples were analysed using protein precipitation followed by HPLC-tandem MS/MS analysis by BIB (Bioanalysis, Immunogenicity and Biomarkers), IVIVT (In Vitro In Vivo Translation), GSK, Ware, Hertfordshire, UK. This method was validated over the range of 0.2 to 500 ng/mL, and the lower limit of quantiﬁcation was 0.2 ng/mL using a 50 µL aliquot of human plasma.

### PK and statistical analysis

This work was performed by CTI SL (Spain). Details are presented in Supplementary Information. Data were analysed using descriptive summary statistics; no formal hypothesis testing was performed. Continuous data were presented with the number of observations, mean values, SD, minimum, median and maximum value. Categorical data were presented as counts and percentages.

## Results

### Demographics and disposition

**Figure 1.**
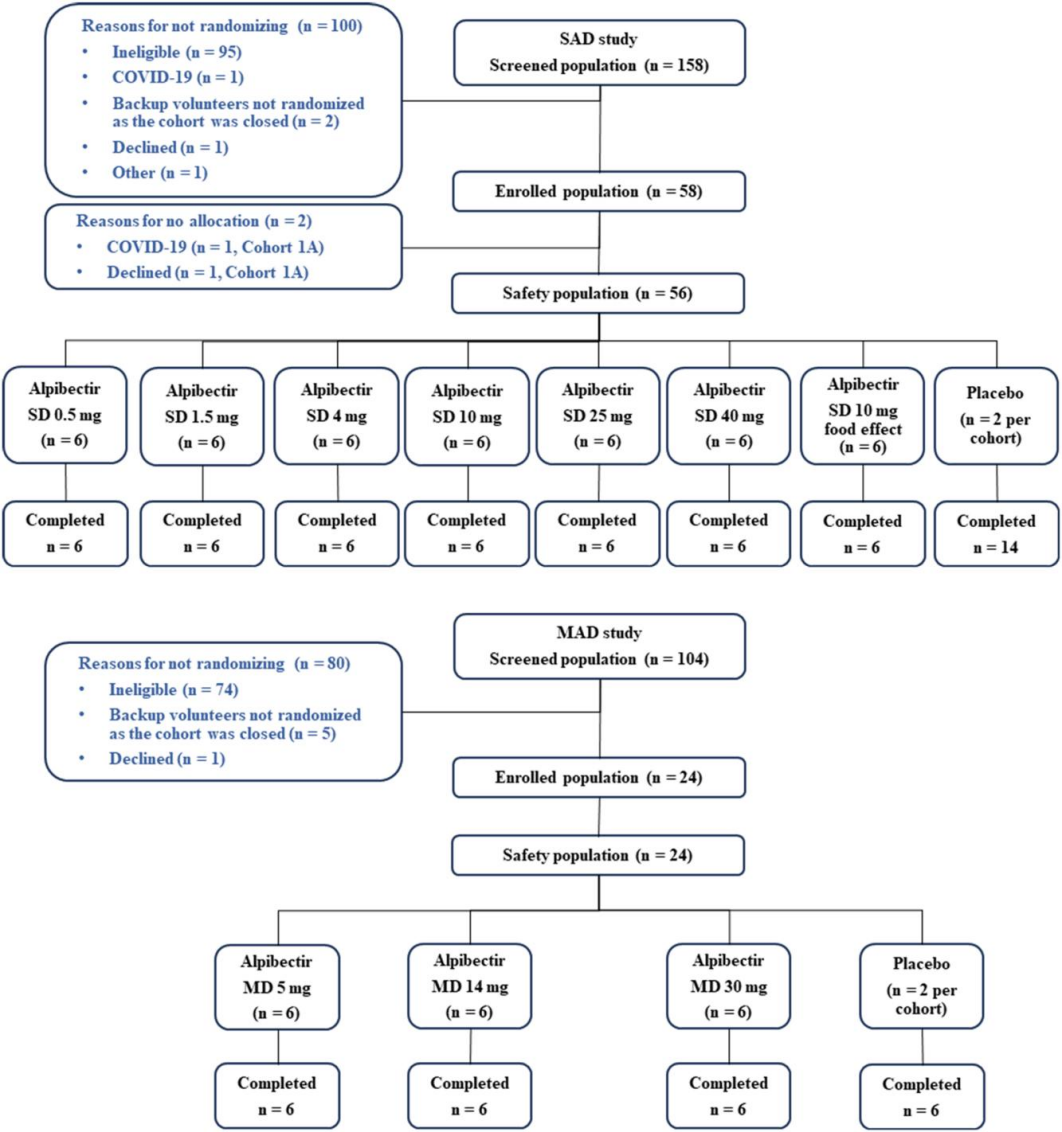
CONSORT diagram showing the screened, enrolled and safety populations of SAD and MAD studies. MD, multiple doses; SD, single dose.

The study, conducted during the COVID-19 pandemic, was started on 27 November 2020, and the last participant had the final study follow-up on 18 April 2022. A total of 80 participants were enrolled in the study, 56 subjects in SAD (seven cohorts of eight participants), and 24 in MAD (three cohorts of eight participants). For each cohort, the participants were randomized in a 3:1 ratio to receive one single (in SAD) or once-daily for 7 days (MAD) doses of alpibectir (six participants/cohort) or placebo (two participants/cohort). No participants discontinued the study treatment (Figure 1).

SAD cohorts 1, 2, 3, 4, 6 and 7 were recruited in site 01 (Sant Pau; *n* = 48), whereas cohort 5 was recruited in site 02 (UEC La Princesa; *n* = 8). MAD was recruited in site 02 only. The demographic characteristics of the participants included in the PK analyses were similar between the SAD and MAD groups. Demographic data are presented in Table 1.

**Table 1.** Baseline demographics of participants in SAD and MAD study

		Value(s) for participants receiving:	
Parameter		SAD (*N* = 56)	MAD (*N* = 24)
Site, *n* (%) of participants	CIM Sant Pau	48 (85.7)	0 (0.0)
	UEC La Princesa	8 (14.3)	24 (100.0)
Sex, *n* (%) of participants	Female	3 (5.4)	0 (0.0)
	Male	53 (94.6)	24 (100.0)
Age, y	Mean (SD)	33 (9)	29 (7)
	Median	31	28
	(Min, max)	(18, 51)	(19, 42)
Race,^a^*n* (%) of participants	American Indian or Alaska Native	6 (10.7%)	18 (75.0%)
	White	50 (89.3%)	6 (25.0%)
Weight, kg	Mean (SD)	74.7 (10.2)	78.2 (10.6)
	Median	72.4	79.2
	(Min, max)	(55.0, 97.0)	(56.1, 95.8)
Height, cm	Mean (SD)	174 (7)	174 (7)
	Median	176	175
	(Min, max)	(160, 187)	(160, 184)
BMI	Mean (SD)	25 (3)	26 (3)
	Median	25	26
	(Min, max)	(19, 30)	(21, 30)

^a^Most of the participants in both sites 01 and 02 have Hispanic or Latin American origin. Site 01 (Sant Pau) considers them as ‘white’ but site 02 (UEC La Princesa) considers them as ‘American Indian or Alaska Native’.

### Safety

No serious AEs, treatment-emergent adverse events (TEAEs) leading to study or treatment discontinuation, or deaths were reported in the study. In the SAD, 56 healthy participants received one single oral dose of alpibectir (*n* = 42) or placebo (*n* = 14). Six (10.7%) participants across groups experienced a total of six TEAEs during SAD, of which two (3.6%) participants reported a TEAE, each possibly related to the study treatment (Table 2). All the six TEAEs were mild/moderate, with no severe TEAEs, and all but one were deemed as resolved within 8 days. Overall, single ascending doses of alpibectir were well tolerated and no clinically relevant safety findings were identified in the participants treated during SAD. In the MAD, 24 healthy participants received seven oral doses of alpibectir (*n* = 18) or placebo (*n* = 6) over a week (q24h). Seven (29.2%) participants across the three cohorts experienced a total of seven TEAEs (Table 3), all of which were assessed as unlikely or unrelated to the study treatment. All the seven TEAEs were mild/moderate, with no severe TEAEs, and all were deemed as resolved within 1–6 days. Overall, multiple ascending doses of alpibectir were well tolerated and no clinically relevant safety findings were identified in the participants who received a dose during MAD.

**Table 2.** Summary of TEAEs ranked by overall frequency and preferred term in SAD study

Preferred term^a^	0.5 mg (*N* = 6)	1.5 mg (*N* = 6)	4 mg (*N* = 6)	10 mg (*N* = 6)	25 mg (*N* = 6)	40 mg (*N* = 6)	10 mg (fed) (*N* = 6)	Total (*N* = 42)	Placebo (*N* = 14)
	*n* (%)	*n* (%)	*n* (%)	*n* (%)	*n* (%)	*n* (%)	*n* (%)	*n* (%)	*n* (%)
Headache	0	0	0	0	1 (16.7)	1 (16.7)^b^	0	2 (4.8)	0
Ligament sprain	1 (16.7)	0	0	0	0	0	0	1 (2.4)	0
Blood alkaline phosphatase increased	0	1 (16.7)^b^	0	0	0	0	0	1 (2.4)	0
Back pain	0	0	0	0	0	0	1 (16.7)	1 (2.4)	0
Neck pain	0	0	0	0	1 (16.7)	0	0	1 (2.4)	0

*n* is the number of participants in each category and percentages are calculated as 100 × (*n*/*N*).

^a^Adverse events were coded using MedDRA (version 23).

^b^Possibly related to study treatment.

**Table 3.** Summary of TEAEs ranked by frequency and preferred term in MAD study

Preferred term^a^	5 mg (*N* = 6)	14 mg (*N* = 6)	30 mg (*N* = 6)	Total (*N* = 18)	Placebo (*N* = 6)
	*n* (%)	*n* (%)	*n* (%)	*n* (%)	*n* (%)
Headache	1 (16.7)	0	1 (16.7)	2 (11.1)	0
Ear pain	1 (16.7)	0	0	1 (5.6)	0
Abdominal pain	0	1 (16.7)	0	1 (5.6)	0
Odynophagia	0	0	0	0	1 (16.7)
Dizziness	0	1 (16.7)	0	1 (5.6)	0
Hand dermatitis	0	0	1 (16.7)	1 (5.6)	0

*n* is the number of participants in each category and percentages are calculated as 100 × (*n*/*N*).

^a^Adverse events were coded using MedDRA (version 23).

In both parts there were no clinically signiﬁcant ﬁndings in clinical laboratory safety test results, vital signs, ECG findings, physical examination ﬁndings or neurological examination ﬁndings.

### PK summary

**Figure 2.**
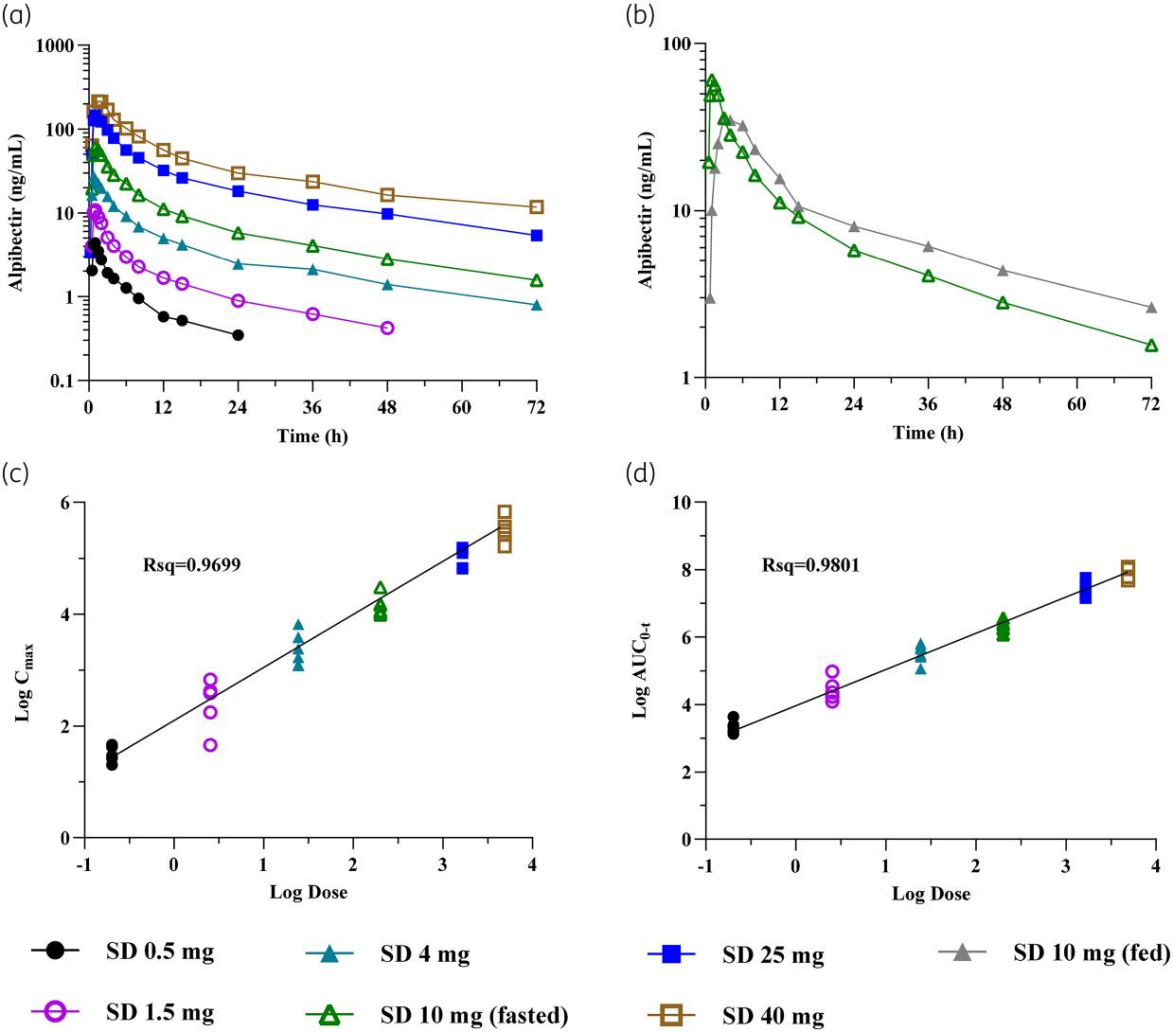
Mean concentration–time profiles, under fasted and fed conditions, and dose proportionality of alpibectir after single ascending doses. (a) (Geometric) mean plasma alpibectir concentration–time plot (semi-log) of all fasted cohorts. (b) (Geometric) mean plasma alpibectir concentration–time plot (semi-log), comparing single doses (SD) of 10 mg in fasted (green) and fed (grey) state. (c) Dose proportionality after single doses in fasted state in terms of *C*_max_, individual participant data. (d) Dose proportionality after single doses in fasted state in terms of AUC_0–*t*_, individual participant data.

In the SAD study, single oral doses of 0.5, 1.5, 4, 10, 25 and 40 mg were administered under fasting conditions whereas the 10 mg dose was also assessed under fed conditions in a separate cohort. PK samples were collected up to 72 h post-dose, and PK parameters derived via non-compartmental analysis are shown in Table 4. Concentration–time profiles as well as the food effect evaluation (10 mg) and an assessment of dose proportionality are depicted in Figure 2.

**Table 4.** Mean PK values for alpibectir after a single dose in SAD study^a^

Cohort	Dose, mg	*N* ^b^	*C*_max_, ng/mL	*T*_max_, h	AUC_0−*t*_, ng·h/mL	*N* ^b^	AUC_0−∞_, ng·h/mL	*t*_1/2_, h
1A	0.5	5	4.48 (15.00)	0.89 (15.86)	28.31 (19.62)	4	33.29 (23.57)	14.21 (44.98)
2A	1.5	6	11.34 (43.82)	0.91 (14.94)	81.96 (32.49)	5	91.86 (33.41)	18.83 (15.03)
3A	4	6	29.01 (29.89)	0.88 (28.97)	250.33 (27.45)	5	270.25 (24.89)	22.58 (30.66)
4A	10	6	63.39 (18.00)	1.04 (31.92)	555.58 (16.48)	6	618.86 (20.93)	23.71 (21.35)
5A	25	6	154.68 (17.73)	1.20 (42.84)	1577.36 (23.36)	5	1818.62 (24.57)	24.78 (14.19)
6A	40	5	246.36 (22.61)	1.53 (52.24)	2607.97 (17.15)	5	3012.03 (15.60)	26.58 (5.78)
7A	10 (fed)	5	52.15 (13.11)	3.87 (51.55)	664.47 (28.42)	5	764.10 (25.89)	25.43 (12.19)

^a^Values represent the geometric mean (coefﬁcient of variation, in %). Parameter values were rounded to two decimal places.

^b^ *N*, number of participants contributing to PK parameter characterization. Reasons for the exclusion of individual participants are described in the ‘Materials and methods’ section.

After single dose administration the mean time to maximum concentration (mean *T*_max_) ranged from 0.88 to 1.53 h across the studied doses under fasting conditions.

The mean elimination half-life (*t*_1/2_) was in the range of 14.21 h to 26.58 h. More specifically, the *t*_1/2_ was 14.21 h and 18.83 h for the two lowest doses (0.5 and 1.5 mg, respectively) and it was higher for doses from 4 to 40 mg (ranging from 22.58 h to 26.58 h) where the *t*_1/2_ appeared to be constant, demonstrating a favourable PK for a daily oral administration at anticipated therapeutic doses.

For the exposures, the observed intersubject variability [expressed as geometric coefficient of variation (CV) in %] was lower than 30% across all doses, with the exception of a slightly increased variability for *C*_max_ and AUC at the dose of 1.5 mg, in which it was more than 30%. The dose proportionality was confirmed across the tested dose range for the AUC to the time of the last quantifiable concentration (AUC_0−*t*_), the slope was 1.031 (R-squared (Rsq) = 0.9801) and the 90% CI included the 1 (0.987–1.075). For *C*_max_ the slope was with a value of 0.915 close to 1 (Rsq = 0.9699). Nonetheless, the 90% CI did not include 1 (0.867–0.963), so dose proportionality could not be concluded.

When alpibectir was administered under fed conditions, the absorption was slower (mean *T*_max_ 3.87 h) leading to a decreased mean *C*_max_ (−17.7%) compared with the fasted condition (Table 4 and Figure 2b). However, the administration of alpibectir with food resulted in an increased mean AUC_0–*t*_ (+19.6%) and AUC_0–∞_ (+20.6%) compared with the fasted condition. The limits of the 90% CI of the geometric means of the fed over fasted ratio (fed/fasted) for AUC_0–*t*_ (93.45%–153.07%), AUC_0–∞_ (93.45%–153.07%), *C*_max_ (68.91%–98.20%) and *t*_1/2_ (88.14%–130.55%) of alpibectir were not within the acceptance limits for bioequivalence (80%–125%), confirming that the food affects the PK of alpibectir.^21^

**Figure 3.**
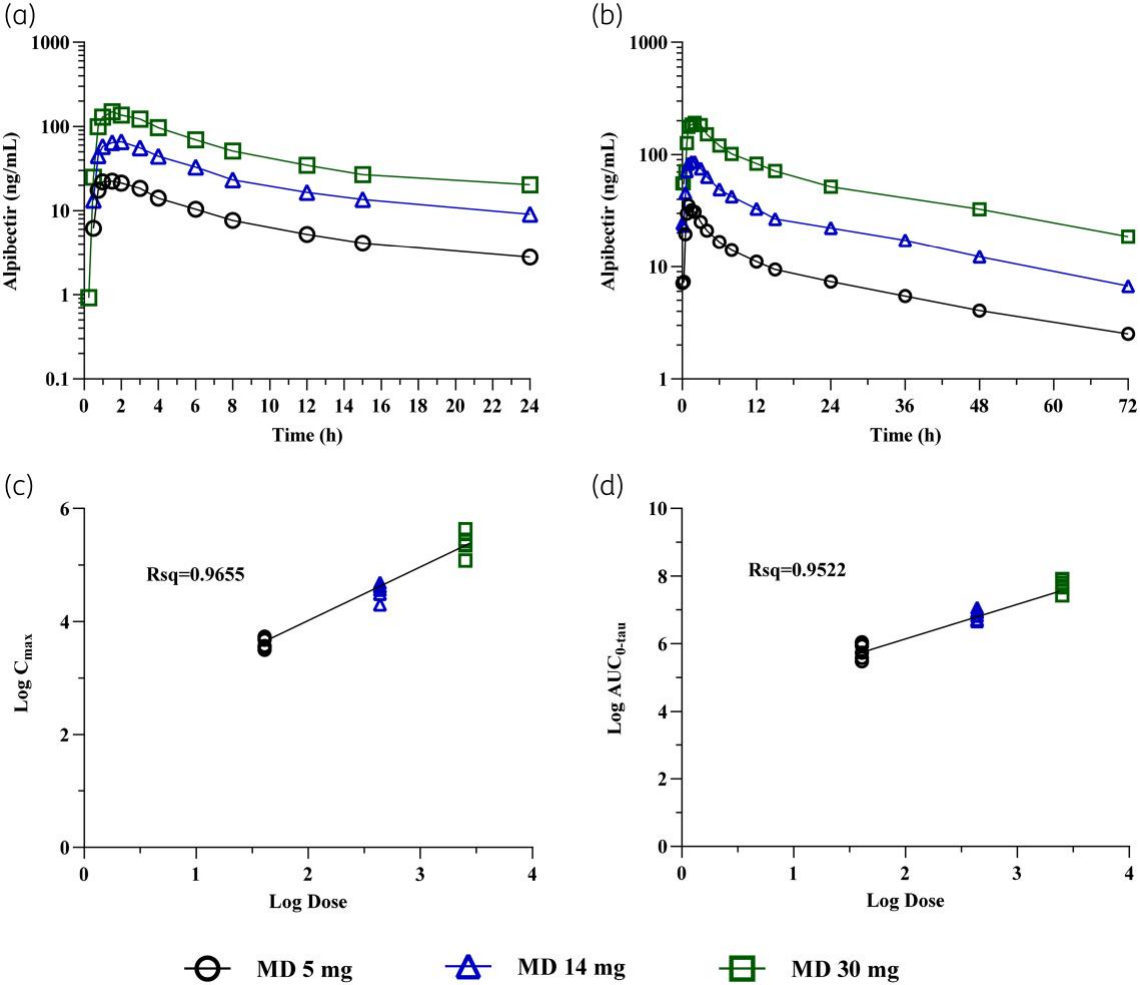
Mean concentration–time profiles and dose proportionality of alpibectir after multiple ascending doses. (a) (Geometric) mean plasma alpibectir concentration–time plot (semi-log) for Day 1. (b) (Geometric) mean plasma alpibectir concentration–time plot (semi-log) for Day 7. (c) Dose linearity after multiple doses in terms of *C*_max_, individual participant data. (d) Dose linearity after multiple doses in terms of AUC_0–tau_, individual participant data. MD, multiple doses.

In the MAD study, the PK of alpibectir after single (Day 1) and multiple doses (Day 7) was evaluated. Daily doses of 5, 14 and 30 mg were administered. PK samples were collected up to 24 h post-dose for Day 1 assessments and up to 72 h post-dose for Day 7 assessments. PK parameters are shown in Tables 5 and 6, and the concentration–time profiles as well as the assessment of dose proportionality are depicted in Figure 3.

**Table 5.** Mean PK values for alpibectir after the first dose (Day 1) and after multiple doses (Day 7) in MAD study^a^

Dose, mg	Day	*N* ^b^	*C*_max_, ng/mL	RAC*_C_*_max_	*T*_max_, h	AUC_0–tau_, ng·h/mL	RAC_AUC0–tau_	*N* ^b^	*t*_1/2_, h
5	1	6	30.37 (24.42)	—	1.22 (73.68)	184.15 (24.57)	—	5	8.32 (13.81)
	7	6	37.38 (9.51)	1.23 (27.48)	1.14 (36.27)	329.93 (24.07)	1.79 (20.72)	4	24.47 (39.17)
14	1	6	75.76 (17.25)	—	1.35 (35.40)	556.16 (17.60)	—	4	8.49 (10.80)
	7	6	94.14 (13.75)	1.24 (23.28)	1.14 (36.27)	947.61 (15.12)	1.70 (7.67)	6	26.10 (23.81)
30	1	6	169.53 (28.59)	—	1.31 (51.59)	1202.13 (28.73)	—	5	9.02 (6.16)
	7	6	217.68 (18.18)	1.29 (23.51)	1.73 (53.25)	2281.30 (17.85)	1.90 (15.67)	6	27.65 (26.97)

^a^Values represent the geometric mean (coefﬁcient of variation, in %). Parameter values were rounded to two decimal places.

^b^ *N*, number of participants. Reasons for the exclusion of extrapolated *t*_1/2_ values from individual participants are described in the ‘Materials and methods’ section.

**Table 6.** *C*_trough_ levels at study Days 5 to 8 in MAD study^a^

	Dose		
Day	5 mg	14 mg	30 mg
Day 5	6.35 (36.62)	20.82 (17.86)	47.72 (18.39)
Day 6	6.10 (64.31)	22.56 (18.34)	53.91 (19.54)
Day 7	7.58 (39.97)	24.77 (17.88)	56.21 (16.71)
Day 8	7.86 (39.52)	22.43 (19.25)	52.39 (18.02)

^a^Values represent the arithmetic mean in ng/mL (coefﬁcient of variation, in percent). Parameter values were rounded to two decimal places. *N* = 6 for all dose cohorts and days.

The *T*_max_ was similar for all tested doses after single and multiple doses. After 7 days of treatment with alpibectir the mean *T*_max_ ranged from 1.14 to 1.73 h. After single and multiple doses, the observed variability was low across all three doses, with geometric mean CV values for *C*_max_ and AUC during a dosing interval (AUC_0–tau_) below 30%. The highest variability was observed for *C*_trough_ for the dose of 5 mg (Table 6).

The elimination half-life (*t*_1/2_) after a single oral dose (Day 1) was similar for all doses, with a mean *t*_1/2_ varying from 8.32 h to 9.02 h. These results must be treated with caution because the PK samples were taken only until 24 h post-dose. The *t*_1/2_ after multiple doses ranged from 24.47 h to 27.65 h. These values were similar to those observed in the SAD study after single dose administration when the elimination was assessed with a more complete PK profile until 72 h.

Dose proportionality was shown after multiple doses over the tested dose range. For AUC_0–tau_, the slope was 1.076 (Rsq = 0.9522) and the 90% CI included the 1 (0.971–1.181). For *C*_max_, the slope was 0.9785 (Rsq = 0.9655) and the 90% CI included the 1 (0.898–1.059).

The assessment of the achievement of steady state was performed using the *C*_trough_ of Day 6, Day 7 and Day 8 (Table 6), also considering that 97% of steady state is reached after five half-lives.^21^ In the first instance, an individual assessment of the achievement of steady state was performed by visual examination. Three participants from the 5 mg cohort and two participants from the 14 mg cohort seemed to have the steady state achievement compromised. Next, a mixed effect model including dose by day interaction was performed to assess the achievement of steady state with a formal statistical analysis. For *C*_trough_, the slope and the 90% CI were 0.018 (−0.014 to 0.050) for the 5 mg dose, −0.001 (−0.031 to 0.029) for the 14 mg dose, and 0.003 (−0.027 to 0.033) for the 30 mg dose, respectively. In all cases, the slope was close to 0% and 90% CI included the 0 indicating achievement of steady state. All participants, except for one (5 mg dose) that had a below limit of quantification value on Day 6, reached the steady state.

The accumulation ratio (RAC) for Day 7/Day 1 for *C*_max_ and AUC_0–tau_ for all the tested doses was calculated. An accumulation of 1.23- to 1.29-fold for *C*_max_ and 1.70- to 1.90-fold for AUC_0–tau_ was observed (Table 5).

## Discussion

In this FIH study, the safety, tolerability, PK and food effect of alpibectir administered as single and multiple oral doses in healthy participants were investigated. Alpibectir up to 40 mg as a single oral dose and up to 30 mg as multiple doses for 7 days was found to be safe and well tolerated. The nature and incidence of AEs did not appear to be dose-related. No dose-limiting toxicities were identiﬁed. There were no clinically signiﬁcant ﬁndings in the serum biochemistry or haematology test results, vital signs, ECGs, or the results of physical or neurological examinations.

Plasma PK of alpibectir showed a rapid absorption, with a mean *T*_max_ of 1–2 h after both single and multiple dose administration of alpibectir. A dose-proportional increase in plasma AUC levels following oral single dose administration from 0.5 to 40 mg was demonstrated. The presence of food affected the PK of alpibectir and resulted in a slower absorption (mean *T*_max_ 3.87 h) and therefore to a decrease in the mean *C*_max_ (−17.7%). Nonetheless, administration with food led to an increased AUC_0–*t*_ (+19.6%) compared with the fasted condition. A dose-proportional increase in plasma *C*_max_ and AUC levels following multiple dose regimens once a day at 5, 14 and 30 mg for 7 days was demonstrated. Accumulation of alpibectir was observed across the tested doses, on average 30% for *C*_max_ and 80% for AUC_0–tau_. Even though the steady state conditions were not met for all individuals on all doses via visual examinations, the statistical analysis demonstrated that steady-state conditions were met after 7 days of dosing.

Recently, we have demonstrated that a small molecule (called SMARt751) that binds VirS regulates the *mymA* operon encoding a monooxygenase that activates Eto in *M. tuberculosis*. SMARt751 boosted the efficacy of Eto *in vitro* and in mouse models of acute and chronic TB. SMARt751 also restored full efficacy of Eto in mice infected with *M. tuberculosis* strains carrying mutations in the *ethA* gene, which cause Eto resistance in the clinic. Interestingly, the SMARt751 has a long-lasting effect *in vitro* and *in vivo*, and its PK does not need to match the Eto PK to reach maximal efficacy.^18^ Alpibectir has the same mode of action as SMARt751 (Edoo *et al.*, unpublished data) and thus is the first example of a drug candidate in development for the treatment of TB that modulates transcription of antibiotic bioactivation. Alpibectir is currently being investigated in a Phase 2a trial in South Africa; an open-label, randomized, two-stage clinical trial is recruiting adults with newly diagnosed, rifampicin- and isoniazid-susceptible pulmonary TB to determine bactericidal activity, PK, safety and tolerability of Eto and alpibectir at various doses (NCT05473195). If alpibectir in combination with a well-tolerated dose of Eto proves to be as bactericidal as the standard Eto dose, the combination may be an attractive candidate in future TB treatment regimens.

Eto is one of the few anti-TB drugs with good penetration into the CSF in patients.^22^ It remains to be demonstrated if alpibectir reaches therapeutic CSF exposures in patients. If confirmed, it could indicate the use of the alpibectir/Eto combination for treatment of the most devastating form of TB, TB meningitis.

## Supplementary Material

dkae107_Supplementary_Data

## Transparency declarations

A.A.G-S., R.M.A.A. and M. Davila have no financial conflicts of interest. E.P., M.J.R. and M.S.M.-M. are employed by and hold stocks in GSK. G.V., G.W.B., S.L.P. and M. Davy are employed or formerly employed by GSK and hold stocks in GSK and Haleon (formerly part of GSK). M.J.R. is also listed as inventor on the patent WO/2019/034700. L.H., M.P., M.G. and G.E.D. are employed by BioVersys AG and/or BioVersys SAS and hold stocks in BioVersys AG. D.O.M. has been consultant or investigator in clinical trials sponsored by the following pharmaceutical companies: Abbott, Alter, Chemo, Cinfa, FAES, Farmalíder, Ferrer, GSK, Galenicum, Gilead, Italfarmaco, Janssen-Cilag, Kern, Normon, Novartis, Servier, Silverpharma, Teva and Zambon. M.Z. is a consultant and investigator for BioVersys clinical trials. The funding agency did not play any decision-making role in the design, execution, analysis or reporting of the research. Medical writing services were provided by Quirino Schefer (regenold GmbH) by delivering the first draft manuscript, intermediate revised versions and by performing the final quality check before submission.
