# Supplementary material for: First-in-human study of alpibectir (BVL-GSK098), a novel potent anti-TB drug
Source: J Antimicrob Chemother. 2024 Apr 24;79(6):1353–61. doi: 10.1093/jac/dkae107 (PMC11144484; doi:10.1093/jac/dkae107)
Supplement: dkae107_Supplementary_Data [file dkae107_supplementary_data.docx]

**Supplementary Information for Phase 1 study of alpibectir by Pieren et *al.***

## Details to study designs and participants

Key exclusion criteria included women of childbearing potential; pregnant or lactating females; individuals with current or chronic history of cardiovascular, renal, hepatic, chronic respiratory or gastrointestinal disease, neurological or psychiatric disorder; ALT, GGT, AST, alkaline phosphatase (ALP) or serum bilirubin levels exceeding the upper limit of normal (ULN), an ECG QT interval corrected for heart rate using Fridericia’s formula (QTcF) of > 450 ms, concomitant use of prescription or nonprescription drugs, history of smoking or heavy alcohol use, and positive HIV or hepatitis B or C virus status. All participants gave written informed consent. The protocol and informed-consent form were approved by the IEC of the Fundació de gestió sanitaria of the Hospital de la Santa Creu I Sant Pau, Barcelona, Spain, prior to inclusion of participants. More details are found in the supplementary information.

The dose selection of alpibectir in the FIH study was based on no-observed-adverse-effect-level (NOAEL) data from the toxicology studies, the predicted PK in humans, and a predicted human therapeutic range of boosting doses of Eto based on efficacy data from mouse models of acute and chronic lung infection.

In SAD, a total of 6 dose levels of alpibectir (0.5, 1.5, 4, 10, 25 and 40 mg) were tested in 7 sequential cohorts. Each cohort was sub-divided into 2 sub-cohorts. The first sub-cohort included 2 sentinel participants: 1 randomized to receive alpibectir and 1 to receive placebo. 72 hours after receiving this single dose, the Sponsor and Principal Investigator (PI) of the given cohort reviewed the safety data before deciding on the inclusion of the remaining 6 participants in the second sub-cohort. The second sub-cohort included the 6 additional remaining participants: 5 randomized to receive alpibectir and 1 to receive placebo. Post-dose, all participants had safety and PK assessments continued for at least 72 h. After completion of each cohort, the safety and PK data were reviewed by the dose escalation committee (DEC) before initiation of the next dose. The decision to move forward to the next dose level in SAD was based on a blinded review of all available safety and tolerability data from at least 6 participants and of PK data from at least 4 participants who had completed assessments through Day 4. Upon completion of the first 6 cohorts of SAD, a data analysis was conducted before starting MAD. The last cohort in SAD investigated the effect of food (high fat, high caloric meal according to FDA guidance^1^) on the PK of alpibectir administering a dose already tested in fasted conditions (10 mg). In MAD, a total of 3 dose levels of alpibectir (5, 14 and 30 mg) were tested in 3 sequential cohorts, each consisting of 8 healthy participants. The cohorts followed the same scheme as for SAD, except the participants received 7 administrations of alpibectir or placebo once daily for 7 days, and the follow-up safety data required for the Sponsor and PI to decide on the initiation of the second sub-cohort was of 5 days instead of 72 hours. Post-dose, all participants were assessed for PK proﬁles on Day 1 (until 24 h) and Day 7 (until 72 h). After completion of each cohort, the blinded data were reviewed by the DEC before initiation of the next dose. The decision to move forward with the next dose level in MAD was based on a blinded review of all available safety and tolerability data from at least 6 participants and of PK data from at least 4 participants who had completed assessments through Day 10. A follow-up visit after completion of the last dose was performed in both parts (Days 10 to 14 in SAD and Days 17 to 20 in MAD).

## Details to PK and statistical analysis

The safety population (defined as all randomised participants who received at least one dose of alpibectir or placebo) was used for demographic and safety analyses, while the PK population (defined as all randomised participants who received at least one dose of alpibectir, met the selection criteria, completed PK sampling of interest, and had sufficient concentrations to obtain reliable estimates of the PK parameters) was used for the PK analyses.

The PK analysis was performed via non-compartmental (NCA) methods using a validated software (Phoenix WinNonlin^®^ Professional, version 8.3, Certara, New Jersey, USA). Derivation of AUCs was made by linear-up/log-down calculation method using the actual sampling times. For PK analysis, summary statistics included mean (arithmetic and geometric), SD, coefficient of variation (arithmetic and geometric), median, minimum, and maximum. Concentrations below limit of quantification (BLOQ) were treated as zero.

For SAD, for the assessment of dose proportionality, a statistical analysis was performed using a power model. The analysis was performed on log_e_ transformed data for C_max_, AUC_0-∞_ and AUC_0‑t_. For each of these parameters a fixed effect model was fitted with log_e_ (dose) as a fixed effect. Estimates of the mean slopes of log_e_ (dose) was reported along with corresponding 90%CIs (slope 1 implied dose proportionality). For MAD, a similar analysis was performed for log_e_ transformed AUC_0- tau_ and C_max_ on Day 7.

The absence of food effect on the PK of alpibectir is established if the 90%CI for the ratio of the population geometric means between fed and fasted conditions [fed/fasted], based on log_e_ transformed data, is not contained in the equivalence limits of 80-125% for AUC_0-t_, AUC_0-∞_, C_max_ and t_1/2_.^1^ An analysis of variance model (ANOVA) was fitted along with 90%CIs using a fixed effects model, with fed/fasted condition as the fixed effect. Point estimates and corresponding 90% CIs were constructed for the comparisons of interest, using the residual variance. These were then back transformed to provide point estimates and corresponding 90%CIs for geometric mean ratios fed/fasted. For T_max_, the analysis was based on descriptive statistics.

The steady state achievement was assessed for each tested dose by using a mixed effect model. The log_e_ transformed C_trough_ (pre-dose samples of Day 5, Day 6, and Day 7 and, additionally the 24 h post-dose sample of Day 7) was used as dependent, the day was fitted as a fixed effect and participant as a random effect. The slope and the 90%CI of the slope were obtained (slope zero or near to zero implied that steady state had been reached). The 90%CI should contain zero. If zero was included in the CI, steady state conditions were reached. As one of the subjects had a BLOQ value at Day 6 that is treated as a zero, geometric means cannot be calculated hence arithmetic means are reported.

No imputation of missing data was done for safety or PK evaluations. There were no positive pre-dose values. The reliability of the PK parameters calculation was assessed, and the following parameters were flagged:

SAD: Two participants were excluded from the PK analysis due to major protocol deviations (cohort 1A: total bilirubin > upper level of normal; cohort 7A: too high alcohol consumption). One participant from cohort 6A showed a plasma concentration BLOQ at 1 h post-dose. Considering that the T_max_ for alpibectir is reached around 1-2 h post-dose, this value was defined as an outlier and the participant was not included in the statistical analyses.

Some PK parameters failed the analysis criteria, were flagged and were not reported. Specifically, the percentage of extrapolated AUC was higher than 20% (1 participant of cohort 1A and 1 of cohort 6A), the R-squared (Rsq) was less than 0.85 (1 participant from cohort 1A and one participant from cohort 5A) and the terminal elimination half-life (t_1/2_) was not determined over a time interval higher than 2 x t_1/2_ (1 participant of cohort 2A, 1 of cohort 3A and 1 of cohort 6A).

MAD: No exclusions were required for the analysis dataset. The elimination half-life (t_1/2_) could not be well characterised in 4 participants after single dose administration and in 2 participants after multiple dose administration. The Rsq was less than 0.85 in 1 participant and the t_1/2_ was not estimated over a time interval higher than 2 x t_1/2_ in 3 participants after single-dose and 2 participants after multiple doses. For 1 participant, the t_1/2_ could not be well characterised after single or multiple doses. The PK parameters failed, the analysis criteria were flagged and excluded from the statistical analyses.

# **REFERENCES**

1. FDA. Food-Effect Bioavailability and Fed Bioequivalence Studies. Guidance for industry. Final version. December 2002.
